# Inconsistent HIV pre‐exposure prophylaxis use and HIV transmission in men who have sex with men (MSM)

**DOI:** 10.1002/jia2.25579

**Published:** 2020-08-26

**Authors:** Daniel Richardson, Jane Shapiro, David A Lewis, Iryna Zablotska, the Western Sydney Sexual Health Centre PrEP Research working group

**Affiliations:** ^1^ Western Sydney Sexual Health Centre Parramatta NSW Australia; ^2^ Marie Bashir Institute for infectious diseases and biosecurity & Westmead Clinical School Faculty of Medicine and Health University of Sydney Westmead NSW Australia

**Keywords:** men who have sex with men, HIV‐pre exposure prophylaxis, HIV prevention, HIV epidemiology, transgender people, sexually transmitted infections/diseases

1

We read with interest Spinelli *et al*.’s study identifying risk factors for HIV acquisition in patients who use HIV Pre‐exposure prophylaxis (PrEP) inconsistently [1]. We concur with these findings and have found some similar associations with inconsistent PREP use in our small cohort of patients in Western Sydney. Between January 2018 and December 2019, five MSM who reported inconsistent PrEP use and then subsequently acquired HIV describe; running out of tablets and failing to get a replacement prescription (2/5, 40%), the break‐down of a regular relationship and no longer perceiving themselves at risk of HIV (1/5, 20%), substance misuse (4/5, 80%), in particular crystal methamphetamine, not having access to being able to afford more PrEP (1/5, 20%). We have also identified some other important factors: 3/5 MSM who used PrEP inconsistently also reported recent sexual contact with a female and self‐identified as “heterosexual.” Men who lead hidden lives, have female partners but who do not disclose that they also have sex with men, are known to take sexual risks and may not be able to consistently use PrEP due to fear of disclosure or not being exposed to health promotion messages designed for MSM [2]. We also agree and have recently shown that some members of the community and healthcare workers remain unfamiliar and doubtful, about the use and effectiveness of on‐demand PrEP despite emerging evidence [3, 4].

The data presented by Spinelli et al. did not examine why some MSM have never used PrEP before acquiring HIV, despite PrEP being available. In our centre, 26 MSM diagnosed with HIV who had never used PrEP were at risk for HIV due to their sexual behaviour. They were commonly born outside Australia (15/26), arriving mainly from Asia, had used recreational drugs (8/26) and also had had sex with women (7/26). Six of these MSM had had prior discussions with a clinician about PrEP, but never used it (3/6 were bisexual, 4/6 reported substance misuse and 4/6 were non‐Australian born).

Western Sydney Sexual Health Centre serves a highly diverse population of Australia with high rates of unemployment and chronic disease; and low average household incomes and educational attainment [5]. We prescribed PrEP to approximately 860 MSM and TGW between January 2018 and December 2019. During this period, there were 30 new HIV diagnoses in MSM and one transgender woman. Eleven out of 31(35%) had a discussion about using PrEP before their HIV diagnosis. Although the numbers of MSM acquiring HIV in our cohort are small, their individual experiences of inconsistent or non‐PrEP use are important and need further evaluation to validate these associations. Understanding why MSM do not access PrEP or use PrEP inconsistently will increase the effectiveness of PrEP.

Ending HIV transmission is possible, but to achieve this goal we have to optimize the impact PrEP can have on the HIV epidemic. There should be no missed opportunities for discussions around PrEP in patient consultations and more effort needs to be placed on exploring the potential barriers faced by patients that prevent them engaging in this safe and cost‐effective biomedical HIV prevention strategy.

### COMPETING INTEREST

None of the authors have declared any competing interest.

### AUTHORS’ CONTRIBUTIONS

DR and IZ designed the study, DR and JS collected and analysed the data, all the authors (DR, JS, DL & IZ) contributed to the final manuscript. All authors have read and approved the final manuscript.

REFERENCES1

Spinelli
MA
, 
Laborde
N
, 
Kinley
P
, 
Whitacre
R
, 
Scott
HM
, 
Walker
N
, et al. Missed opportunities to prevent HIV infections among pre‐exposure prophylaxis users: a population‐based mixed methods study, San Francisco, United States. J Int AIDS Soc. 2020;23:e25472.3229433810.1002/jia2.25472PMC71592492

Ragonnnet‐Cronin
M
, 
Hue
S
, 
Hodcroft
EB
, 
Tostevin
A
, 
Dunn
D
, 
Fawcett
T
, et al. Non disclosed men who have sex with men in UK transmission networks: phylogenetic analysis of surveillance data. Lancet HIV. 2018;5(6):309–16.10.1016/S2352-3018(18)30062-6298932443

Power
M
, 
Lewis
D
, 
Richardson
D
. On‐demand HIV pre‐exposure prophylaxis: what are the healthcare workers’ barriers to delivery in a culturally and linguistically diverse population?
Sex Transmit Infect. 2020; [Epub ahead of print]. 10.1136/sextrans-2019-054393
319114274

Molina
JM
, 
Ghosn
J
, 
Algarte‐Génin
JM
, 
Rojas Castro
D
, 
Béniguel
L
, 
Pialoux
G
, et al; ANRS Study Group. Incidence of HIV‐infection with daily or on‐demand PrEP with TDF/FTC in Paris area. Update from the ANRS Prévenir Study. Abstract TuAC0202. Oral abstracts of the 10th iAS Conference on HiV Science, 21–24 July 2019, Mexico City, Mexico. J Int AIDS Soc. 2019;22:e25327.313390045

King
M
, 
Usherwood
T
, 
Brooker
R
, 
Reath
J
.Supporting primary Health care providers in Western Sydney areas of socio‐economic disadvantage. Canberra: Australian Primary Health Care Research Institute; 2016 [cited 2020 Mar 17]. Available from: https://rsph.anu.edu.au/files/Supporting%20PHC%20Providers%20in%20western%20Sydney%20areas%20of%20socio‐sconomic%20disadvantage.pdf

